# Omega-3 for the Prevention of Alcohol Use Disorder Relapse: A Placebo-Controlled, Randomized Clinical Trial

**DOI:** 10.3389/fpsyt.2022.826448

**Published:** 2022-04-08

**Authors:** Renata Pauluci, Ana Regina Noto, Daniela Fernandez Curado, Miguel Siqueira-Campos, Andréia Gomes Bezerra, José Carlos Fernandes Galduróz

**Affiliations:** Departamento de Psicobiologia, Universidade Federal de São Paulo, São Paulo, Brazil

**Keywords:** omega-3 fatty acids, alcohol dependence, relapse, inpatients, neuroprotection

## Abstract

**Background:**

Recent studies have sought to identify the possible benefits of the intake of omega-3, an important component of neuronal membranes, for the treatment of alcohol use disorder.

**Aim:**

The objective of the present study was to evaluate whether omega-3 supplementation is protective against alcohol use disorder relapse after hospital discharge.

**Methods:**

A randomized, double-blind, placebo-controlled study was carried out with severe alcohol dependence. Male inpatients were randomized to treatment with omega-3 (*n* = 59) or placebo (*n* = 52) for 3 months, participants were assessed after discharge at 1 month (T1), 2 months (T2), 3 months (T3), and 6 months (T4) with assessments made using self-report instruments. The primary outcomes were the possible reduction with assessments made using self-report instruments. The primary outcomes were the possible reduction in the number, intensity of relapses, amount of consumption in each relapse and number of days of consumption during relapses; as secondary outcomes were assessed symptoms of anxiety, depression, degree of dependence, compulsion, and craving.

**Results:**

The groups were similar regarding consumption amount parameters and propensity to relapse; however, an effect of treatment with omega-3 was found on the number of days of drinking at 2 months [*B* = 0.65 (0.09; 1, 21), *p* = 0.01] and 3 months [*B* = 2.6 (1.61; 3.58), *p* < 0.001] after discharge, favoring the intervention group. The effect was not maintained at follow up of 6 months. No differences were found in psychiatric symptoms and severity of addiction.

**Conclusion:**

Despite the major limitations of the present study, the group that received omega-3 had a lower number of days of consumption of standard doses of alcohol in the evaluations of 60 and 90 days after discharge. More robust studies are needed to confirm or refute these findings. Brazilian Registry of Clinical Trials: n° RBR-48mkgz7 (URL: https://ensaiosclinicos.gov.br/rg/RBR-48mkgz7).

## Introduction

Alcohol use disorder is associated with several negative health outcomes, including morbidity, mortality and disability (1). To date, there are few drugs approved for its pharmacological treatment. In the United States, the Food and Drug Administration (FDA) has approved only disulfiram, naltrexone and acamprosate. In the European Union, the European Medicines Agency (EMA) also approved gamma-hydroxybutyrate and nalmefene (2).

The effects of approved drugs are limited and only modestly superior to those of placebo (3). The challenges to the treatment of alcohol use disorder include recurrent relapses, with rates ranging from 40 to 60% within 1 year (4). Relapse rates vary widely in clinical studies; some studies show a short-term remission rate of 20 to 50%, while others indicate that between 20 and 80% of individuals who achieve short-term remission relapse in the long term (5). Thus, there is an urgent need for new therapeutic alternatives.

Polyunsaturated fatty acids (PUFAs), mainly ômega-3 and ômega-6, are being studied as a possible adjuvant in alcoholism disorders treatment (6). In an animal model, ômega-3 reduced anxiety-like behaviors in newborn rats that previously received ethanol (7). Shi et al. (8) described that fish oil reduced preference to ethanol chamber in a mice model of alcohol dependence. In the same study, was showed that, in the nucleus accumbens, fish oil prevented ethanol-induced dendritic morphological changes. A clinical study of our group intended to analyze the possible effect of PUFAs in alcohol use disorder. Although the participants decrease dependence symptoms, no difference was found comparing PUFAs and placebo groups (9). Two aspects could be highlighted in this previous study: the capsules were composed by omega-3 and omega-6, and, the participants could continue drinking alcohol in their routines (9).

Omega-3 and omega-6 are both constituents of cell membranes, however, have some different functions. Omega-6 has gammalinolenic acids (GLA), dihomogamalinolenic acids (DGLA) and arachidonic acids (AA) such metabolities and it is involved in increase cytokine production (10). Omega-3 has metabolites eicosapentaenoic acids (EPA) and docosahexanoic acids (DHA) and it is involved of inhibition of IL-1 and IL-6 cytokines, that is, have an anti-inflammatory properties, and inflammation is one of the pathophysiological mechanisms involved in neuropsychiatric and neurodegenerative disorders (11). Moreover omega-3 are related to the functions of some neurotransmitters (10, 12) that certainly are involved to alcohol dependence mechanisms. It is discussed that supplementation with omega-3 could improve the functioning of the mesolimbic, mesocortical and serotonergic-dopaminergic pathways (13).

Ethanol could inhibit the activity of enzymes of metabolic pathways by which linoleic acid become omega-6 and alpha-linolenic acid become omega-3 (14, 15). It is described the association between low plasmatic levels of docosahexaenoic acid and relapses in substance abusers (16) showing that alcohol can decrease the availability of these substances in the organism.

The hypothesis of the present study is that by supplementing an important component of neuronal membranes with anti-inflammatory properties, the omega-3 could control inflammatory mechanisms associated with alcohol use disorder. Additionally, considering for this study only inpatients, that is, without usual alcohol use pattern, might be consider that omega-3 stabilizes neuronal membranes, reorganizing the functioning of the mesolimbic pathway (reinforcement pathway). Therefore, it was evaluated whether this specific PUFA, the omega-3 supplementation, can decrease the number, intensity, and duration of relapses in severe alcohol-dependent individuals after discharge.

## Materials and Methods

### Study Design

This was a randomized, double-blind, placebo-controlled study with male participants admitted to a hospital or therapeutic community specialized in dependence treatment in Brazil. To be admitted in these health services, all patients had been evaluated by a psychiatry that diagnosed the alcohol dependence.

The study comprised two groups that participants were randomly assigned: control (placebo) and intervention (omega-3). The respective substances were used for 90 days, and the intervention was initiated after a period of detoxification of 7 days (this period was determined by Institution). During the study intervention period, the participants maintained the usual treatments recommended by the institution, both pharmacological [sertraline-50 mg/day, chlorpromazine-100 mg/day, diazepam-10 mg/day and thiamine (vitamin B1)-300 mg/day] all medicines by oral route; and non-pharmacological (psychotherapy-once a week, occupational therapy-three times a week, and physical activities-twice a week). These non-pharmacological therapies were offered to all participants by the Institution during inpatients, but it was not a mandatory activity.

The evaluation was performed before the start of the intervention (T0), and after discharge at 1 month (T1), 2 months (T2), 3 months (T3), and 6 months (T4) with assessments made using self-report instruments to determine the number, intensity, and duration of relapses. Data were collected from June 2018 to January 2021, following the CONSORT guidelines (17). This study protocol was approved by the Research Ethics Committee of the Federal University of São Paulo (#0965/2017). Starting in 2020, with the beginning of the COVID-19 pandemic, new participants stopped being recruited, and evaluations of the already included participants were made by phone. The trial practical was registered after the data collection was completed on the Brazilian Registry of Clinical Trials: n° RBR-48mkgz7^1^.

### Participants

The inclusion criteria were as follows: age between 18 and 70 years, male sex, diagnosis of alcohol use disorder in accordance with the DSM-5 criteria (18) and assessment of the degree of dependence using the Short Alcohol Dependence Data (SADD) questionnaire (19).

The exclusion criteria were a history of allergy to fish and a history of severe diseases, that is, with high risk of mortality, of hepatic, cardiovascular, renal, pulmonary, endocrine, neurological or other origins. Individuals with psychiatric disorders with psychotic symptoms, such as schizophrenia, or illicit drug addiction were also not included in the study.

All participants signed an informed consent form before inclusion in the study and for all participants were guaranteed to leave the research if they wished. If this occurred, it was counted as a dropout.

### Intervention

Capsules had an identical external appearance and were manipulated by a brazilian pharmacy (Famader Farmácia de Manipulação Ltda, São José dos Pinhais, PR, Brazil). The omega-3 capsules contained 1 g of fish oil, with at least with 12% of docosa-hexaenoic acid (DHA) and 18% of eicosapentaenoic acid (EPA) in their composition. The placebo capsules contained 1 g of rice oil. The participants were instructed to ingest 1 capsule every 8 h, that is, 3 capsules a day, for 3 months. The capsules had no smell, however, all participants were instructed to take them before meals, because the participants in the intervention group could experience “fish oil burps.” However, in the present study, no participant reported this complaint.

The capsules were administered for 90 days, and the dosage chosen for this study was based on the dosage considered safe, according to the FDA, and consisted of the same dose recommended for the prevention of cardiovascular events, that is, 3 g a day (20). After hospital discharge, in the monthly evaluation, treatment adherence was controlled by counting the remaining capsules. At this meeting, the capsules for the next month were delivered for participants.

### Randomization and Blinding

The research coordinator generated a sequence 1:1 using a software random.org program and the allocation was codified by a letter (A or B). Only this researcher had the information to which group the participant was allocated. The bottles of omega-3 or placebo were coded in the Department of Psychobiology by a researcher independent of the interviews with the participants.

Study investigators, attending care teams and the participants were blinded to treatment allocation. At the end of the study, the secret was revealed only to the researcher who conducted the statistical analysis.

### Clinical Evaluation and Instruments Used

The participants were subjected to a clinical interview that included their medical history and to a physical examination. The participants were also asked to provide the contact information of a family member or friend for support and interviews) after discharge from the hospital.

The questionnaires and instruments used to assess primary and secondary outcomes are described below.

### Primary Outcomes

#### Timeline Followback

The TLFB allows retrospectively estimating the consumption of alcohol and other drugs. Quantitative estimates and consumption variables can be used to measure the change in substance use levels over an established period of time (21). In the present study, during each clinical evaluation, participants self-reported the number of alcoholic drinks they had ingested in the last 30 days. The following variables were calculated using the instrument: consumption as a binary variable (presence or absence) indicating whether there was at least one episode of alcohol consumption in the 30 days prior to the evaluation; doses consumed, i.e., total alcoholic drinks consumed in the last 30 days; number of days of consumption, i.e., number of days in the period (last 30 days) in which there was alcohol consumption, regardless of the amount; and latency, i.e., the number of days without consumption until a first episode of alcohol use occurred. Participants who did not drink in the 30-day period prior to the evaluation were classified as having the maximum possible latency (31 days).

### Secondary Outcomes

#### Beck Scales

These instruments were used to evaluate symptoms of depression (Beck Depression Inventory—BDI) and anxiety (Beck Anxiety Inventory—BAI) (22).

#### Obsessive-Compulsive Drinking Scale

The OCDS is a self-report scale comprising 14 items that measure the compulsion to drink (craving), attempts to control drinking and obsessive-compulsive components (23).

#### Penn Alcohol Craving Scale

The PACS is a brief, five-item, self-report scale that measures craving for alcohol. The first three items address the frequency, intensity and duration of thoughts regarding alcohol consumption. The fourth item asks the participant to rate how difficult it is to resist drinking, and the fifth and last item asks the participant to rate the “average” alcohol craving they experienced in the previous week (24).

#### Short Alcohol Dependence Data

The SADD has been adapted and validated for the Brazilian context. This is a self-report scale that measures the degree of alcohol dependence. The final score might indicate: 1–9 low dependence, 10–19 medium dependence, and 20 or greater high Dependence (19).

### Sample Size Calculation

The sample size was calculated using the software GPower 3.1, based on ANOVA for repeated measures, effects between subjects, a significance level of 5%, a power of 80%, a correlation between repeated measures of 0.5, two groups, four measurements and an expected small effect size (0.2), yielding 126 participants. The estimate of the effect size was based on two previous studies that tested the efficacy of omega-3 in the treatment of alcohol dependence (9) and tobacco dependence (25).

To account for possible losses to follow-up or refusals to participate, the baseline calculation of the sample size was increased by 46%, the highest rate of loss/abandonment obtained in the study by Zaparoli et al. (25). An additional 10% was added to the calculation to account for the greater complexity of the analyses, resulting in a total sample size of 208 participants required for the present study.

Due to the COVID-19 pandemic, we had to interrupt data collection, resulting in a smaller sample size than originally planned.

### Statistical Analysis

The analyses were performed in R software version 4.0.2 using the glmmTMB package (26). First, the sample was characterized at baseline, comparing outcome scores and demographic characteristics using the *t*-test for continuous variables and the chi-squared (χ^2^) test for categorical variables, in order to evaluate the success of randomization. Simple randomization method was used to random assignments to groups, a random number table was generated by computer program^2^.

The analytical approach of the present study was “intention-to-treat,” including patients randomly allocated to one of the experimental groups in the analyses regardless of the number of follow-up responses. The intention-to-treat approach is an analytical procedure that includes in the analyses both the patients who completed the treatment and those who dropped out. Therefore, it avoids analyzing only those participants who have completed the treatment. By including those who have discontinued their participation throughout the process, it indirectly corrects the analysis for subjective aspects such as motivation and perception of benefit from the treatment.

Generalized mixed models were used to evaluate the effectiveness of the intervention across time. In all models, group, time and the interaction between group and time were included as fixed effects. The ID of the participants was added as a random effect. We considered sociodemographic variables that differed at baseline as control variables. The distribution of the dependent variables was chosen based on their nature.

The outcomes amount of alcohol consumed in the period, intensity of cravings, dependence severity, obsessive and compulsive behaviors, anxiety and depression were modeled following a linear distribution. The presence or absence of relapse was considered a binomial distribution. The number of days of consumption and latency to the first relapse were count variables; therefore, a Poisson distribution was used.

## Results

**FIGURE 1:**
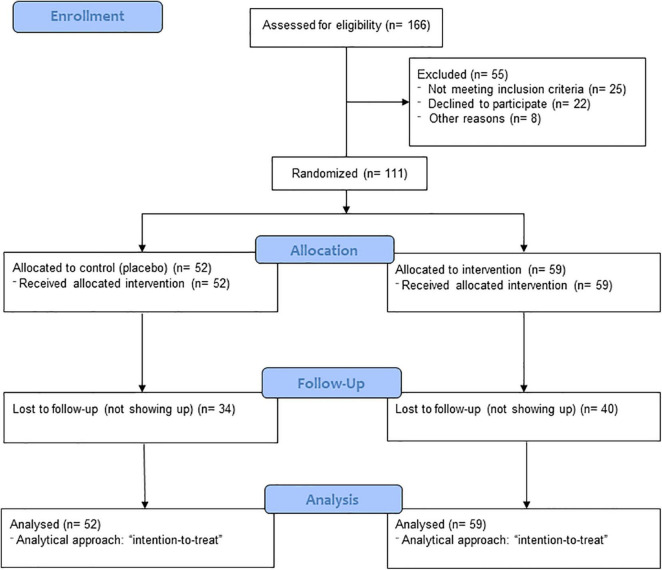
Study flowchart.

A total of 166 participants were invited to participate in the study. Of these, only 111 agreed or were able to participate and were randomly allocated to one of the two treatment groups. The complete flowchart of the study is shown in Figure 1.

The characteristics of the participants at baseline are provided in Table 1. The sample consisted entirely of men aged between 21 and 68 years (mean = 41.6; SD = 9.7). Seventy-one participants reported not having a job (64%), 22 (20%) reported having no monthly income, and 52% had an income of less than two times the minimum wage. Only 18% reported being married; however, most reported having children (73%). As the control and intervention groups showed differences in education level and marital status at baseline, these variables were controlled in subsequent analyses. As explained in the CONSORT guidelines (17), these differences can be considered to have occurred randomly and not as a result of selection bias between the groups, to which participants were randomly assigned. The clinically significant outcomes were equivalent at baseline, as shown in Table 2.

**TABLE 1:** Sociodemographic characteristics at baseline for the total sample and by experimental group.

	Total sample (*n* = 111)	Omega-3 (*n* = 59)	Placebo (*n* = 52)	*p*
Age—mean ± (SD)	41.61 (9.76)	41.02 (9.65)	42.29 (9.93)	0.50
**Monthly family income (US$)—n (%)**				
No income	22 (20)	13 (22)	9 (17)	0.23
Up to the minimum wage	36 (32)	16 (27)	20 (38)	
1–2 times the minimum wage	22 (20)	15 (25)	7 (13)	
2–3 times the minimum wage	14 (13)	7 (12)	7 (13)	
3–5 times the minimum wage	11 (10)	7 (12)	4 (8)	
5–10 times the minimum wage	2 (2)	0 (0)	2 (4)	
10–20 times the minimum wage	2 (2)	0 (0)	2 (4)	
More than 20 times the minimum wage	0 (0)	0 (0)	0 (0)	
**Education level—*n* (%)**				
No education	0 (0)	0 (0)	0 (0)	0.01*
Incomplete primary education	35 (32)	25 (42)	10 (19)	
Complete primary education	20 (18)	6 (10)	14 (27)	
Incomplete secondary education	18 (16)	8 (14)	10 (19)	
Complete secondary education	29 (26)	18 (31)	11 (21)	
Incomplete higher education	6 (5)	2 (3)	4 (8)	
Complete higher education	3 (3)	0 (0)	3 (6)	
**Marital status—n (%)**				
Married	20 (18)	6 (10)	14 (27)	0.04*
Widowed	4 (4)	2 (3)	2 (4)	
Divorced	15 (14)	6 (10)	9 (17)	
Single	71 (64)	44 (75)	27 (52)	
Previous hospitalizations—*n* (%)	53 (48)	29 (49)	24 (46)	0.81
Smoker—*n* (%)	53 (48)	29 (49)	24 (46)	0.40

*p < 0.05 in the comparisons between the placebo and omega-3 groups; the t-test was used for continuous variables, and the chi-squared test was used for categorical variables.

**TABLE 2:** Mean ± (SD) for the outcomes related to substance use, craving, anxiety and depression at baseline (T0) for the total sample and by group.

Outcome	Total sample (*n* = 111)	Omega-3 (*n* = 59)	Placebo (*n* = 52)	*P*
Number of days of consumption^a^ in the last 30 days	3.48 (6.23)	3.93 (6.81)	2.97 (5.54)	0.49
Alcoholic drinks consumed^b^ in the last 30 days	13.95 (35.59)	10.98 (33.87)	10 (28.73)	0.87
Craving intensity (PACS)^b^	12.06 (8.28)	12.22 (8.06)	11.87 (8.60)	0.82
Compulsive behaviors (OCDS)^b^	9.83 (5.92)	9.42 (6.04)	10.29 (5.82)	0.46
Obsessive behaviors (OCDS)^b^	18.03 (8.20)	17.53 (8.31)	18.59 (8.13)	0.51
Dependence severity (SADD)^b^	26.12 (10.89)	26.19 (10.70)	26.04 (11.21)	0.94
Anxiety (BAI)^b^	17.14 (14.18)	15.52 (13.17)	19.04 (15.20)	0.21
Depression (BDI)^b^	18.95 (11.33)	19.23 (11.68)	18.61 (11.00)	0.78

*The analysis used Poisson^a^ or linear^b^ regression models to compare the groups at baseline. PACS, Penn Alcohol Craving Scale; OCDS, Obsessive-Compulsive Drinking Scale; SADD, Short Alcohol Dependence Data; BAI, Beck Anxiety Inventory; BDI, Beck Depression Inventory.*

Tobacco use was reported by 48% of the participants, who smoked an average of 28 cigarettes per day.

More than half of the participants (53%) reported having previously been hospitalized, with a mean of 4.3 (± 6.9) hospitalizations over the lifetime.

**FIGURE 2:**
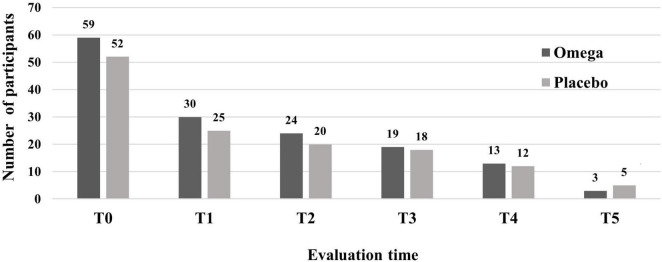
Adherence of participants to interventions in the omega-3 and placebo groups. During hospitalization: T0 = screening, T1 = 1 month, T2 = 2 months, T3 = 3 months; Follow-up after starting treatment: T4 = 6 months. The number of participants in each evaluation is shown in absolute numbers above each bar. There were no significant differences between groups [χ^2^ (4) = 0.14, *p* = 0.99].

A total of 111 participants were randomly allocated to one of the groups, resulting in 59 participants in the omega-3 group and 52 participants in the placebo group. The rates of adherence to interventions were not significantly different between the groups [χ^2^ (4) = 0.14, *p* = 0.99] and are shown in Figure 2.

### Primary Outcomes

**FIGURE 3:**
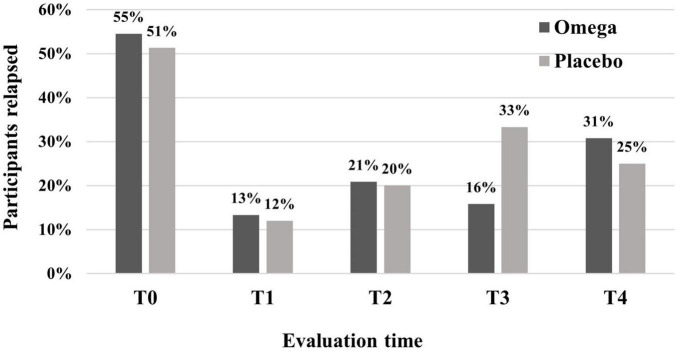
Percentage of participants who had at least one drinking episode (lapse or relapse), as evaluated by the TLFB, in the omega-3 and control groups across time. No significant differences were found between the groups [Wald χ^2^ (4) = 1.66, *p* = 0.80].

There were no effects of treatment with omega-3 on the propensity to have a drinking episode (lapse or relapse) in the month prior to the evaluation [Wald χ^2^ (4) = 1.66, *p* = 0.80]. The percentage of participants in each group who had at least one drinking episode in each evaluated period is shown in Figure 3.

No differences were found between the groups in the amount of alcohol consumed in any of the evaluated periods [Wald χ^2^ (4) = 1.78, *p* = 0.78]. However, the omega-3 group consumed alcohol on fewer days than did the control group at T2, 2 months after the start of treatment [*B* = 0.65 (0.09; 1.21), *p* = 0.01], and at T3 [*B* = 2.6 (1.61; 3.58), *p* < 0.001], 3 months after the start of treatment, with a progressive increase in the difference between these two temporal measures. Both differences were consistent [Wald χ^2^ (4) = 29.89, *p* < 0.001].

**FIGURE 4:**
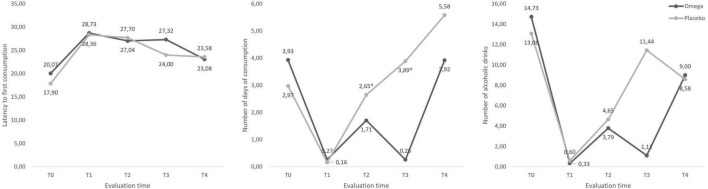
Mean number of days of alcohol consumption, alcoholic drinks consumed in the period (1 month) and latency to the first consumption episode across time for the two groups. There was a significant difference in the number of days of alcohol consumption, favoring the group that received omega-3 [Wald χ^2^ (4) = 29.89, *p* < 0.001]. There was also lower numerical alcohol consumption in favor of the omega-3 group, at the same time periods; however, this difference did not represent a significant effect [Wald χ^2^ (4) = 1.78, *p* = 0.78]. A numerically higher latency was also observed in the omega-3 group at T3, but no effects were identified [Wald χ^2^ (4) = 1.06, *p* = 0.90].

The latency to a first drinking episode did not differ between the groups [Wald χ^2^ (4) = 2.70, *p* = 0.61]. The results of the regression models predicting the TLFB outcomes are shown in Table 3. Figure 4 shows line graphs representing the mean number of days of consumption, amount of alcohol consumed and latency to the first drinking episode in the period.

**TABLE 3:** Non-standardized regression coefficients (followed by 95% confidence intervals) of the mixed models predicting outcomes related to alcohol consumption measured by the TLFB.

	Relapse^1^	Alcoholic drinks consumed^2^	Number of days of consumption^2^	Latency^2^
	Exp. B (95% CI)	B (95% CI)	B (95% CI)	B (95% CI)
**Time (reference = Screening)**				
First visit	0.07 (0.02; 0.30)	−15.80 (−26.47; −5.13)	−2.77 (−3.50; −2.04)	0.43 (0.32; 0.54)
Second visit	0.12 (0.03; 0.48)	−11.62 (−23.08; −0.17)	−1.03 (−1.41; −0.65)	0.37 (0.25; 0.49)
Third visit	0.08 (0.02; 0.41)	−14.49 (−26.88; −2.09)	−2.7 (−3.60; −1.79)	0.36 (0.23; 0.49)
Follow-up (6 months)	0.2 (0.04; 0.97)	−7.13 (−21.43; 7.18)	0.08 (−0.30; 0.47)	0.19 (0.04; 0.35)
**Group (reference = Omega)**				
Placebo	0.74 (0.24; 2.32)	−2.2 (−12.62; 8.21)	−0.52 (−1.53; 0.49)	−0.11 (−0.38; 0.17)
**Time × Group**				
First visit	0.96 (0.13; 7.34)	2.1 (−13.55; 17.75)	−0.45 (−1.7; 0.8)	0.08 (−0.09; 0.25)
Second visit	1.11 (0.16; 7.92)	2.43 (−14.36; 19.21)	0.65 (0.09; 1.21)*	0.11 (−0.07; 0.29)
Third visit	3.33 (0.43; 26.05)	11.64 (−6.16; 29.43)	2.6 (1.61; 3.58)*	0.01 (−0.18; 0.2)
Follow-up (6 months)	0.81 (0.08; 7.95)	0.19 (−20.44; 20.82)	0.27 (−0.27; 0.81)	0.13 (−0.10; 0.35)

*Generalized mixed models considering (1) a binomial distribution with exponential coefficients and (2) a Poisson distribution.*

*In all models, time, group and interaction were included as fixed effects, the ID of the subject was included as a random effect, and education level and marital status were added as control variables.*

*p < 0.05.

*TLFB, Timeline Followback.*

### Secondary Outcomes

Table 4 shows the results of the regression models predicting the other self-report measures related to alcohol use disorder used in the present study. No differences were found between the placebo and omega-3 groups across time regarding cravings [Wald χ^2^ (4) = 1.31, *p* = 0.86], obsessive behaviors [Wald χ^2^ (4) = 6.35, *p* = 0.17], compulsive behaviors [Wald χ^2^ (4) = 2.21, *p* = 0.70] and dependence severity [Wald χ^2^ (4) = 0.95, *p* = 0.92]. There were also no effects of the intervention on symptoms of anxiety [Wald χ^2^ (4) = 3.86, *p* = 0.42] and depression [Wald χ^2^ (4) = 1.43, *p* = 0.84] (Table 5).

**TABLE 4:** Non-standardized regression coefficients (followed by 95% confidence intervals) of the mixed models predicting outcomes related to alcohol dependence.

	Cravings	SADD	Obsessive	Compulsive
	B (95% CI)	B (95% CI)	B (95% CI)	B (95% CI)
**Time (reference = Screening)**				
First visit	−5.62 (−8.39; −2.85)	−10.77 (−14.92; −6.62)	−3.71 (−5.50; −1.93)	−12.17 (−14.71; −9.62)
Second visit	−7.25 (−10.26; −4.23)	−15.56 (−19.91; −11.2)	−5.67 (−7.64; −3.7)	−14.11 (−16.92; −11.31)
Third visit	−6.72 (−10.02; −3.41)	−19.35 (−24.12; −14.58)	−6.57 (−8.69; −4.45)	−15.41 (−18.41; −12.4)
Follow-up (6 months)	−4.85 (−8.99; −0.70)	−17.21 (−23.17; −11.25)	−6.74 (−9.29; −4.19)	−13.76 (−17.38; −10.15)
**Group (reference = Omega-3)**				
Placebo	−1.33 (−4.06; 1.4)	−2.55 (−6.76; 1.66)	0.09 (−1.87; 2.06)	0.19 (−2.52; 2.9)
**Time × Group**				
First visit	−2.26 (−6.34; 1.83)	−1.1 (−7.12; 4.92)	−3.22 (−5.88; −0.57)	−2.77 (−6.54; 1.01)
Second visit	−0.29 (−4.77; 4.2)	−2.47 (−8.91; 3.98)	−1.8 (−4.69; 1.09)	−0.9 (−5; 3.21)
Third visit	−0.17 (−4.91; 4.58)	0.93 (−5.93; 7.79)	−0.52 (−3.63; 2.59)	−0.64 (−5.07; 3.78)
Follow-up (6 months)	−0.93 (−6.7; 4.85)	−0.16 (−8.5; 8.17)	−0.5 (−4.11; 3.11)	−2.2 (−7.34; 2.93)

*Mixed linear models.*

*In all models, time, group and interaction were included as fixed effects, the ID of the subject was included as a random effect, and education level and marital status were added as control variables.*

*SADD, Short Alcohol Dependence Data.*

**TABLE 5:** Non-standardized regression coefficients (followed by 95% confidence intervals) of the mixed models predicting anxiety and depression.

	BAI	BDI
	
	B (95% CI)	B (95% CI)
**Time (reference = Screening)**		
First visit	−7.17 (−11.22; −3.12)	−8.70 (−12.08; −5.32)
Second visit	−13.25 (−17.73; −8.77)	−14.25 (−17.91; −10.59)
Third visit	−14.46 (−19.27; −9.65)	−14.62 (−18.54; −10.7)
Follow-up (6 months)	−13.03 (−19.01; −7.06)	−13.66 (−18.51; −8.81)
**Group (reference = Omega-3)**		
Placebo	1.85 (−2.6; 6.3)	−1.22 (−5.24; 2.79)
**Time × Group**		
First visit	−5.37 (−11.45; 0.7)	−0.08 (−5.06; 4.9)
Second visit	−0.53 (−7.24; 6.18)	2.98 (−2.42; 8.38)
Third visit	0.5 (−6.42; 7.43)	0.97 (−4.73; 6.66)
Follow-up (6 months)	−2.42 (−10.78; 5.93)	0.54 (−6.31; 7.4)

*Mixed linear models.*

*In all models, time, group and interaction were included as fixed effects, the ID of the subject was included as a random effect, and education level and marital status were added as control variables.*

*BAI, Beck Anxiety Inventory; BDI, Beck Depression Inventory.*

## Discussion

In this study, the daily administration of omega-3 for a period of 3 months did not reduce relapse in patients with alcohol use disorder. This result is similar to that found in a previous study conducted with outpatients (9).

However, in the present study, there was a progressive reduction in the number of days on which alcohol was consumed among the participants who received the intervention, 2 and 3 months after the start of omega-3 supplementation, after discharge. This effect was not maintained at follow up of 6 months. In contrast to the aforementioned study, the intervention in this study was initiated in an environment protected from alcohol (hospitalization). It is known that the use of alcohol alters the absorption and metabolism of essential nutrients such as PUFAs; therefore, the initiation of the intervention in an alcohol-free environment may have contributed to this result (20). Perhaps this is the greatest importance of the present study: considering that alcohol dependence is a chronic disease, studies with longer duration of treatment should be considered.

On the other hand, there was also a numerical difference between the groups in the latency and alcoholic drinks consumed, but these differences were not statistically significant; importantly, the small sample size may not have given power to the analyses, resulting in a type 2 error. Such results were not found in a previous study (9).

Regarding the other parameters related to alcohol consumption, propensity to relapse, dependence severity and psychiatric symptoms, there was no significant difference between the groups. In contrast, a previous study reported a reduction in anxiety levels in patients with substance use disorder who received omega-3 supplementation (27).

To date, there are no statistically significant data published on the effectiveness of omega-3 in the treatment of relapses of alcohol use disorder. A systematic review analyzed 2,231 studies involving omega-3 and alcohol dependence and, despite obtaining inconclusive results in clinical trials, found improvements in preclinical studies at the behavioral, cellular, and molecular level (6).

Instead, a narrative review suggested that the ingestion of the omega-3 metabolite, docosahexaenoic acid (DHA), in childhood and adolescence has an important role in the neurodevelopment of the prefrontal cortex, an area involved with impulse control, contributing to decrease vulnerability to alcohol dependence (28). In addition, a clinical trial evaluated the effect of omega-3 supplementation (1 g of fish oil daily) for 3 weeks in a group of patients hospitalized for alcohol use disorder (29). A reduction in cortisol levels and a decrease in stress symptoms were observed, but the post-discharge relapse rate was not evaluated.

Other studies have been conducted involving patients with substance use disorder such as cocaine (27) and smoking (25). Daily administration of 2,250 mg of EPA and 500mg of DHA for 3 months decreased anger and anxiety scores in a group of cocaine addicts. The increase in EPA and DHA was evaluated in plasma. An increase in EPA levels was associated with a lower anxiety score, while an increase in DHA was associated with lower anger scores (27).

A cross-sectional study with smokers showed that smokers have lower serum concentrations of DHA compared to non-smokers. In that same study, n-3 supplementation (389.52 mg DHA/632.97mg EPA) for 3 months had no significant difference in serum nicotine dose or cigarette consumption (25).

In addition, another clinical trial with heavy smokers in which a dose of 5 g of omega-3 per day was used (a dose greater than that considered safe by the FDA) for 3 months showed a statistically significant reduction in the number of cigarettes (30).

The interpretation of the results should be evaluated within the context of the limitations of the study. It was not possible to reach the calculated sample size of 208 participants. Initially, there were difficulties in establishing partnerships with inpatient psychiatric institutions. Beginning in March 2020, during the quarantine period due to the COVID-19 pandemic, researchers were banned from entering partner institutions; inclusion of new participants was interrupted; and the assessment scales began to be applied by phone. However, because recruitment and most of the interventions occurred before the pandemic, there was probably no impact on the results. Other important point is that the patients have been hospitalized for at least a week (alcohol detoxification) when we can have access to them. Therefore, when we applied the baseline TLFB, we obtained that participant’s standard alcohol use from just 3 weeks prior to admission. This must have affected the baseline consumption data collected, for this reason the baseline consume seems lower than in patients with alcohol abuse disorder.

It was not possible to differentiate between lapse and relapse in data collection. Relapse is when the patient returns to the typical pattern of use, while lapse is a single, isolated episode of consumption (31). This information was collected as “an episode of consumption,” as shown in Figure 3.

The plasma level of PUFAs of the participants was not measured, making it impossible to analyze the omega-6/omega-3 ratio and potentially generating diet-related bias because eating habits and physical activity vary greatly among individuals. Some researchers argue that it is necessary to analyze the ratio between omega-6 and omega-3 PUFAs because omega-6 metabolites (except gamma-linolenic acid) have inflammatory properties, while omega-3 has anti-inflammatory properties (10). There is an association between the increase in this ratio and the incidence of several diseases because an imbalance in favor of a high amount of omega-6 is highly proinflammatory (32). Inflammation is one of the pathophysiological mechanisms of psychiatric diseases (11). The current Western diet, rich in processed foods, contains high levels of omega-6 and low levels of omega-3, leading to disproportional levels of these PUFAs. In recent decades, there has been an increase in omega-6 intake at the expense of omega-3, increasing from 1:1 in the early stages of evolution to 20:1 currently. Some studies claim that the ideal omega-6 to omega-3 ratio is approximately 4:1 (33).

To date, there are no statistically significant data in the literature indicating the efficacy of omega-3 in the avoidance of alcohol use disorder relapse. A systematic review analyzed 2,231 studies involving omega-3 and alcohol dependence and, despite the inconclusive results obtained in clinical trials, improvements at the behavioral, cellular and molecular levels were found in preclinical studies (6). In turn, a narrative review suggested that the intake of the omega-3 metabolite docosahexaenoic acid (DHA) in childhood and adolescence plays an important role in the neurodevelopment of the prefrontal cortex, an area involved in impulse control, contributing to a decrease in vulnerability to alcohol dependence (28). In addition, a clinical trial evaluated the effect of omega-3 supplementation (1 g of fish oil daily) for 3 weeks in a group of patients hospitalized due to alcohol use disorder. A reduction in cortisol levels and a decrease in stress symptoms were observed, but the rate of relapse post discharge was not evaluated (29). Other studies with patients with disorders due to the use of other substances, such as cocaine (27) and smoking (25), showed, respectively, a reduction in anxiety symptoms and decreased serum levels of DHA. In addition, another clinical trial with heavy smokers using a dose of 5 g of omega-3 per day (dose higher than that considered safe by the FDA) for 3 months showed a statistically significant reduction in the number of cigarettes smoked (30).

## Conclusion

The present study found no positive effects of omega-3 administration on the prevention of relapse in patients with alcohol use disorder. However, the group that received omega-3 reported fewer number of days of alcohol consumption 60 days after the start of omega-3 supplementation, an effect that remained until 90 days of intervention. Omega-3 treatment seems promising, but further studies with more robust samples are needed to assess its benefits and durability of effect. These studies could perform the blood dosage of PUFAs and analyze the omega-6/omega3 ratio, optimizing the ideal dose for each individual and determining the treatment time. Complementary studies with animal models could also contribute to understanding the mechanisms involved.

## Data Availability Statement

The raw data supporting the conclusions of this article will be made available by the authors, without undue reservation.

## Ethics Statement

The studies involving human participants were reviewed and approved by Research Ethics Committee of the Federal University of São Paulo (#0965/2017). The patients/participants provided their written informed consent to participate in this study.

## Author Contributions

AN and JG: conceptualization, resources, and supervision. RP, AB, MS-C, and JG: methodology. DC: formal analysis. RP, AN, DC, MS-C, AB, and JG: investigation, writing—review and editing. AB: data curation. RP, AB, and JG: writing—original draft preparation. AB and JG: visualization. All authors have agreed to have their name added to the manuscript, contributed to the work and are familiar with the primary data, read the final version of the manuscript and approved its content.

## Conflict of Interest

The authors declare that the research was conducted in the absence of any commercial or financial relationships that could be construed as a potential conflict of interest.

## Publisher’s Note

All claims expressed in this article are solely those of the authors and do not necessarily represent those of their affiliated organizations, or those of the publisher, the editors and the reviewers. Any product that may be evaluated in this article, or claim that may be made by its manufacturer, is not guaranteed or endorsed by the publisher.

## Footnotes

1
https://ensaiosclinicos.gov.br/rg/RBR-48mkgz7

2
www.randomizer.org
